# Quantitative susceptibility mapping as an imaging biomarker for Alzheimer’s disease: The expectations and limitations

**DOI:** 10.3389/fnins.2022.938092

**Published:** 2022-08-05

**Authors:** Yuto Uchida, Hirohito Kan, Keita Sakurai, Kenichi Oishi, Noriyuki Matsukawa

**Affiliations:** ^1^Department of Neurology, Nagoya City University Graduate School of Medical Sciences, Nagoya, Japan; ^2^The Russell H. Morgan Department of Radiology and Radiological Science, Johns Hopkins University School of Medicine, Baltimore, MD, United States; ^3^Department of Integrated Health Sciences, Nagoya University Graduate School of Medicine, Nagoya, Japan; ^4^Department of Radiology, National Center for Geriatrics and Gerontology, Ōbu, Japan

**Keywords:** Alzheimer’s disease, biomarker, imaging, MRI, quantitative susceptibility mapping

## Abstract

Alzheimer’s disease (AD) is the most common type of dementia and a distressing diagnosis for individuals and caregivers. Researchers and clinical trials have mainly focused on β-amyloid plaques, which are hypothesized to be one of the most important factors for neurodegeneration in AD. Meanwhile, recent clinicopathological and radiological studies have shown closer associations of tau pathology rather than β-amyloid pathology with the onset and progression of Alzheimer’s symptoms. Toward a biological definition of biomarker-based research framework for AD, the 2018 National Institute on Aging–Alzheimer’s Association working group has updated the ATN classification system for stratifying disease status in accordance with relevant pathological biomarker profiles, such as cerebral β-amyloid deposition, hyperphosphorylated tau, and neurodegeneration. In addition, altered iron metabolism has been considered to interact with abnormal proteins related to AD pathology thorough generating oxidative stress, as some prior histochemical and histopathological studies supported this iron-mediated pathomechanism. Quantitative susceptibility mapping (QSM) has recently become more popular as a non-invasive magnetic resonance technique to quantify local tissue susceptibility with high spatial resolution, which is sensitive to the presence of iron. The association of cerebral susceptibility values with other pathological biomarkers for AD has been investigated using various QSM techniques; however, direct evidence of these associations remains elusive. In this review, we first briefly describe the principles of QSM. Second, we focus on a large variety of QSM applications, ranging from common applications, such as cerebral iron deposition, to more recent applications, such as the assessment of impaired myelination, quantification of venous oxygen saturation, and measurement of blood– brain barrier function in clinical settings for AD. Third, we mention the relationships among QSM, established biomarkers, and cognitive performance in AD. Finally, we discuss the role of QSM as an imaging biomarker as well as the expectations and limitations of clinically useful diagnostic and therapeutic implications for AD.

## Introduction

Alzheimer’s disease (AD) is the most common cause of dementia (Scheltens et al., 2016). The pathological hallmarks include deposition of extracellular β-amyloid (Aβ) aggregates as senile plaques and intracellular hyperphosphorylated tau aggregates as neurofibrillary tangles, along with neuronal loss and glial activation (Serrano-Pozo et al., 2011). Over a long period, researchers and clinical trials have mainly focused on Aβ pathology, which is hypothesized to be one of the most important factors in AD pathogenesis. However, recent clinicopathological and radiological data suggest that tau pathology, not Aβ pathology, closely links with onset and progression of Alzheimer’s symptoms (Brier et al., 2016; Aillaud and Funke, 2022) though the relationship and interplay between Aβ and tau pathologies remain controversial (Pourhamzeh et al., 2021). Toward a biological definition of biomarker-based research framework for AD, the 2018 National Institute on Aging–Alzheimer’s Association working group has updated the ATN classification system (Jack et al., 2018), whose measures have different roles for definition and staging: A: Aβ biomarkers determine whether an individual is in the Alzheimer’s continuum; T: pathological tau biomarkers determine if an individual in the Alzheimer’s continuum has AD; and N: neurodegenerative biomarkers determine the staging severity of the Alzheimer’s continuum.

In addition to these traditional pathological features, iron deposition has attracted the attention of researchers as a new biomarker reflecting disease severity in AD. Histochemical and histopathological studies have shown evidence of altered iron metabolism and accumulation in AD brain tissues, with iron colocalizing in senile plaques and neurofibrillary tangles (Tao et al., 2014). These abnormal proteins bind ferric iron and reduce it to the redox-active form, ferrous iron, which reacts with hydrogen peroxide to generate hydroxyl radicals, leading to the ferroptosis pathway (Sayre et al., 2000; Everett et al., 2014; Conrad et al., 2016). Studies in animal models of AD have reported that brain iron chelation can abolish this iron-mediated pathomechanism, reducing downstream oxidative stress and neurofibrillary tangle formation (Smith et al., 1997; Guo C. et al., 2013). Therefore, iron may have a synergistic role with Aβ and tau proteins in key pathophysiological processes leading to AD pathogenesis.

Using advanced imaging techniques, human subjects were investigated *in vivo* to determine whether their brain iron levels would be altered. Quantitative susceptibility mapping (QSM) has recently become more popular as a non-invasive magnetic resonance technique with which to quantify local tissue susceptibility with high spatial resolution; this technique is sensitive to the presence of iron (Liu et al., 2009; Shmueli et al., 2009; de Rochefort et al., 2010). In this review, we focused on the associations of established pathological biomarkers for AD with cerebral iron deposition using a conventional QSM technique, as well as more complicated QSM applications, such as an assessment of impaired myelination, quantification of venous oxygen saturation, and measurement of blood–brain barrier function in clinical settings for AD.

## Principles of quantitative susceptibility mapping

### History of quantitative susceptibility mapping

Magnetic susceptibility between tissues has been utilized as a new type of contrast in magnetic resonance imaging (MRI), which differs from proton density, T1-, and T2-weighted imaging. The phase signals from materials with different magnetic susceptibilities compared with their neighboring tissues are formed by dipole interactions. The phase image itself is unavailable without post-processing for phase unwrapping, which is performed to deconvolute the dynamic range of −π to π, and background field removal for susceptibility differences at tissue-air boundaries. Thus, phase imaging provides a unique contrast between gray matter, white matter, iron-laden tissues, venous blood vessels, and other tissues with biologically specific magnetic susceptibilities that differ from those of background tissues (Liu et al., 2009; Shmueli et al., 2009; de Rochefort et al., 2010). Susceptibility-weighted imaging (SWI) is a precursor post-processing technique for QSM that uses the phase as a means of enhancing susceptibility differences (Haacke et al., 2004). Since its development in the mid-1990s (Haacke et al., 1995), SWI has been used in diverse clinical settings, such as in the identification of cerebral microbleeds (Akter et al., 2007; Greenberg et al., 2009; Barnes et al., 2011; Goos et al., 2011; Cheng et al., 2013; Guo L. F. et al., 2013; Linn, 2015; Shams et al., 2015), acute ischemic stroke (Hermier and Nighoghossian, 2004; Tong et al., 2008; Santhosh et al., 2009; Tsui et al., 2009; Chalian et al., 2011; Kesavadas et al., 2011; Baik et al., 2012; Kao et al., 2012; Fujioka et al., 2013; Lou et al., 2014; Meoded et al., 2014; Verma et al., 2014; Luo et al., 2015), vascular malformations (Essig et al., 1999; Choi and Mohr, 2005; Jagadeesan et al., 2011), and magnetic resonance venography (Reichenbach et al., 1997, 1998, 2001; Reichenbach and Haacke, 2001; Neelavalli et al., 2014). However, these approaches are qualitative in nature as SWI is calculated by the summation of magnitude and homodyne-filtered phase signals (Liu et al., 2017). This limitation is currently being addressed with the development of the QSM technique (Liu et al., 2015), which provides a quantitative measure of magnetic susceptibility and has been useful for statistical image analyses (Eskreis-Winkler et al., 2017).

### Acquisition and reconstruction protocols for quantitative susceptibility mapping

A 3D gradient-recalled echo sequence with full flow compensation is generally used to acquire QSM data, as this sequence can account for the flow-induced phase shift and capture reliable phase information (Schenck, 1996; Xu et al., 2014). The properties of the gradient echo signal phase images produced by a clinical 3 Tesla MRI scanner are highly dependent on the imaging parameters (Haacke et al., 2015). Multiple echo sequences can acquire phase data more effectively than single-echo sequences. The phase value is dependent on the frequency map and echo time, and it achieves optimal phase contrast and maximal signal-to-noise ratios when the echo time is equal to the T2* value on a specific pixel (Wu et al., 2012). As the optimal echo time is usually different in various tissue types due to the variety of T2* values, it is necessary to combine the frequency map at each echo time based on the weighted averages of the T2* values. The parallel imaging technique is turned on to reduce the scan time as long as the magnitude and phase images are properly reconstructed (Vinayagamani et al., 2021). A high-resolution whole-brain acquisition of 6–12 min is typically implemented. Low spatial resolution and small brain coverage worsen the accuracy of susceptibility values (Karsa et al., 2019).

Susceptibility map reconstruction consists of several post-processing steps, which include phase unwrapping, background field removal, and dipole inversion. As the phase data are limited to the dynamic range from −π to π, a phase unwrapping algorithm is required to calculate the frequency map (i.e., total field map) (Robinson et al., 2017; Karsa and Shmueli, 2019). Then, the background field caused by the air-tissue interface is removed from the total field map to separate the tissue-generated field map (Liu T. et al., 2011; Sun and Wilman, 2014; Zhou et al., 2014; Kan et al., 2016, 2018; Özbay et al., 2017). The susceptibility map is finally reconstructed from the tissue-generated field map using dipole inversion processing (Liu et al., 2009; de Rochefort et al., 2010; Wharton et al., 2010; Wei et al., 2015; Liu Z. et al., 2018; Polak et al., 2020). The mean susceptibility value of the cerebrospinal fluid in the lateral ventricles is usually defined as a zero reference, given that it is essentially water and contains negligible iron (LeVine et al., 1998; Haacke et al., 2015).

Based on the concept described above, we adopt a gradient echo sequence with the following parameters from our previous study (Uchida et al., 2019): number of echoes: 5; minimal first echo time: 6.4 ms; Δ echo time: 6.4 ms; repetition time: 36 ms, flip angle: 15; field of view: 192 × 192 × 160 mm^3^; matrix: 192 × 192; and slice thickness: 1 mm, yielding an iso-voxel resolution of 1 mm^3^ on a 3 Tesla MRI scanner. The QSM reconstruction algorithm includes the Laplacian-based algorithm (Bagher-Ebadian et al., 2008), variable-kernel sophisticated harmonic artifact reduction for phase data to remove the background field owing to the existence of an air–tissue interface (Kan et al., 2016, 2018; Özbay et al., 2017), and improved sparse linear equations and least-squares techniques (Li et al., 2015; Wei et al., 2015). Note that different approaches have been proposed for each post-processing step, which influences the accuracy of the magnetic susceptibility values and the edge of the brain mask (Haacke et al., 2015). Details of MRI acquisition parameters and postprocessing techniques in QSM studies for AD continuum subjects are summarized in Table 1 (Acosta-Cabronero et al., 2013; Hwang et al., 2016; Moon et al., 2016; van Bergen et al., 2016b,^2018^; Ayton et al., 2017; Kim et al., 2017; Meineke et al., 2018; Tiepolt et al., 2018; Chen et al., 2020; Kagerer et al., 2020; Kan et al., 2020; Tuzzi et al., 2020; Cogswell et al., 2021; Ravanfar et al., 2021; Uchida et al., 2022b).

**TABLE 1:** Overview of MRI acquisition parameters and postprocessing techniques in QSM studies for AD continuum subjects.

Study	MRI scanner	Field strength	Head coil	Voxel size (mm)	TE (ms)	ΔTE (ms)	Number of echoes	Acquisition sequence	Phase unwrapping	Background field removal	Dipole inversion
Acosta-Cabronero et al. (2013)	Trio, Siemens	3T	12-channel phased-array head coil	1 × 1 × 2	20	NA	NA	FLASH	Laplacian-based	NA	MEDI
van Bergen et al. (2016b)	Achieva, Philips	7T	32-channel receive array head coil	0.5 × 0.5 × 0.5	6	6	3 (2 echoes used)	GRE	Laplacian-based	V-SHARP	LSQR
Moon et al. (2016)	Signa, GE	3T	8-channel head coil	0.94 × 0.94 × 2	3.5	4.09	8	GRE (based on SWAN)	Magnitude-guided	PDF	MEDI
Hwang et al. (2016)	Achieva, Philips	3T	8-channel SENSE head coil	0.63 × 0.63 × 1.26	34	NA	1	GRE	Quality-guided	PDF	MEDI
Ayton et al. (2017)	Trio, Siemens	3T	12-channel head coil	0.93 × 0.93 × 1.75	20	NA	NA	GRE	Laplacian-based	V-SHARP	iLSQR
Kim et al. (2017)	Achieva, Philips	3T	8-channel SENSE head coil	0.68 × 0.68 × 2.2	3.4	6	7	3D FFE	NA	PDF	MEDI
Tiepolt et al. (2018)	Magnetom, Siemens	7T	24-channel head coil	0.7 × 0.7 × 0.7	10	NA	NA	GRE	SDI QSM processing algorithm		
van Bergen et al. (2018)	Signa, GE	3T	8-channel head coil	1 × 1 × 1	6	4	6	bipolar GRE	Laplacian-based	SHARP	iLSQR
Meineke et al. (2018)	Ingenia, Philips	3T	32-channel RF receive head-coil	0.6 × 0.6 × 2	3.5	4	7	GRE	JEDI QSM processing algorithm		
Chen et al. (2020)	Achieva, Philips	3T	NA	1 × 1 × 1	6	6	5	GRE	Best-path based	V-SHARP	
Kan et al. (2020)	Ingenia, Philips	3T	20-channel receiver head–neck coil	1 × 1 × 1	6	6.2	5	MP-QSM	Laplacian-based	V-SHARP	iLSQR
Kagerer et al. (2020)	Signa, GE	3T	8-channel head coil	1 × 1 × 1	6	4	6 (3 echoes used)	GRE	Laplacian-based	V-SHARP	LSQR
Tuzzi et al. (2020)	Siemens	9.4T	31-channel receive RF array head coil	0.13 × 0.13 × 0.61	16.5	NA	1	GRE	Laplacian-based	RE-SHARP	iLSQR
Cogswell et al. (2021)	Prisma, Siemens	3T	NA	0.52 × 0.52 × 1.8	6.7	3.9	5	GRE	STI Suite QSM processing algorithm		LSQR
Uchida et al. (2022b)	Ingenia, Philips	3T	32-channel head coil	1 × 1 × 1	6	6.2	5	MP-QSM	Laplacian-based	V-SHARP	iLSQR

AD, Alzheimer’s disease; FFE, fast field-echo; FLASH, fast low-angle shot; GRE, gradient (recalled) echo; iLSQR, iterative LSQR; JEDI, joint background-field removal and segmentation-enhanced dipole inversion; LSQR, sparse linear equation and least-squares; MEDI, morphology-enabled dipole inversion; MP-QSM, magnetization-prepared spoiled turbo multiple gradient echo sequence with inversion pulse for QSM; MRI, magnetic resonance imaging; NA, not applicable; PDF, projection onto dipole fields; QSM, quantitative susceptibility mapping; RE-SHARP, Regularization-enabled SHARP; SDI, superfast dipole inversion; SHARP, Sophisticated Harmonic Artifact Reduction for Phase; SWAN, susceptibility weighted angiography; TE, echo time; V-SHARP, Variable-radius SHARP.

## Clinical applications of quantitative susceptibility mapping

### Quantification of iron content

Quantifying tissue iron concentration *in vivo* is the best clinical application of QSM to understand the role of iron in the pathophysiology of neurological diseases associated with abnormal iron distribution. The mean susceptibilities of the bulk tissue in deep gray matter nuclei have been validated using total iron content *ex vivo* or *in vitro* and measured using various modalities, including synchrotron X-ray fluorescence iron mapping (Zheng et al., 2012, 2013), atomic absorption spectrometry (House et al., 2007), and inductively coupled plasma mass spectrometry (Langkammer et al., 2010, 2012b). The challenge is that the estimation of iron concentration in white matter regions is less accurate and more complex due to the counteracting contribution from diamagnetic myelinated neuronal fibers that confounds the interpretation (Langkammer et al., 2012a). Another challenge is the estimation of age-related iron changes in deep gray matter nuclei and myelin changes in white matter regions (Bilgic et al., 2012; Keuken et al., 2017; Lee et al., 2018; Zhang et al., 2018; Ning et al., 2019). In order to draw any conclusions regarding the presence of abnormal iron accumulation, it will be necessary to know the range and variation of normal susceptibilities for all ages. A 4D developmental QSM atlas serves as a template for studying brain iron deposition and myelination/demyelination during normal aging and in various brain diseases (Zhang et al., 2018).

### Assessment of myelination

Evaluating white matter alterations in the AD brain, in addition to gray matter alterations, has been of great interest. The magnetic susceptibility of white matter is mainly influenced by iron and myelin components (Shmueli et al., 2009; Haacke et al., 2010). Human brain myelination changes over the entire lifespan (Lebel et al., 2012); it is prominent in the brain development that occurs during early life (Deoni et al., 2012; Lee et al., 2018), in the normal aging processes that occur later in life (Lee et al., 2012; Zhang et al., 2018), and during pathological demyelination (Liu C. et al., 2011; Langkammer et al., 2013; Cao et al., 2014). As white matter fiber bundles are myelinated, susceptibility values are more diamagnetic (Li et al., 2014; Zhang et al., 2018). Therefore, QSM provides valuable information regarding the temporal and spatial patterns of brain myelination and demyelination. Further research is warranted to quantify the changes in myelin content in various physiological and pathological conditions such as brain development, aging, neurodegenerative diseases, and demyelinating diseases (Vinayagamani et al., 2021).

### Measuring venous oxygen saturation

In addition to gray and white matter structures, blood vessels in the brain are also key factors in AD pathogenesis. Close monitoring of central venous oxygenation serves as a novel biomarker for studying cerebral hemodynamics (Eskreis-Winkler et al., 2017), which can aid in understanding the pathophysiology of vascular disorders in which blood oxygen supply is impaired. Differential diagnosis between AD and vascular cognitive impairment is quite difficult because their pathophysiologies are overlapped as well as their concurrence. Brain oxygen extraction fraction (OEF) is differentially altered by AD and vascular cognitive impairment (Jiang et al., 2020). QSM has recently been used to measure venous oxygen saturation; hence, the cerebral metabolic rate for oxygen and OEF can be calculated (Gauthier and Hoge, 2012; Fan et al., 2015; Zhang et al., 2015; Kudo et al., 2016; Uchida et al., 2022a). Briefly, the OEF calculation from the QSM is expressed as follows:


$$O\,E\,F=\frac{\Delta \,\chi \times P_{v}}{\Delta \,\chi_{d\,o}\times H\,c\,t}$$

where Δχ is the susceptibility difference between the vein and surrounding brain tissue, Δχ_*do*_ is the difference in susceptibility per unit of hematocrit between fully deoxygenated and fully oxygenated blood, *Hct* is each subject’s hematocrit, and *P*_*v*_ is a correction factor for the partial volume effects that was defined based on the simulated calculation (Kudo et al., 2016). Rapid acquisition of magnetic susceptibility and evaluation of venous oxygen saturation can aid in the determination of predictors for progressive ischemic regions in urgent care settings (Kan et al., 2017, 2019). QSM-derived OEF map shows the area of the penumbra as an indicator of brain cell viability. It has been reported that brain tissues with increased OEF values can predict ischemic penumbral tissues based on diffusion-perfusion mismatch areas defined by a dynamic susceptibility contrast (Uchida et al., 2022a).

### Biomarker for neurodegenerative diseases

**FIGURE 1:**
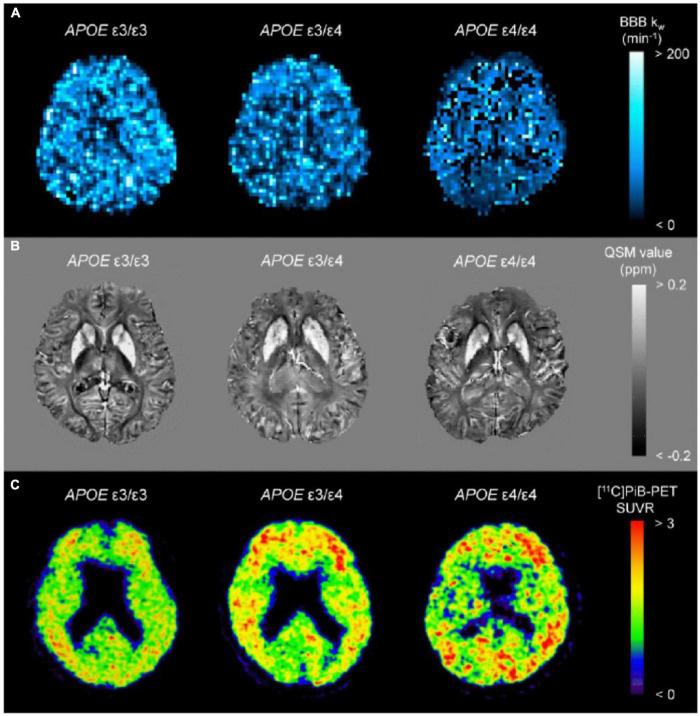
Representative images from BBB k_*w*_ map **(A)**, QSM **(B)**, and [^11^C]PiB-PET SUVR **(C)** from a APOE ε4 non-carrier (ε3/ε3), a heterozygote (ε3/ε4), and a homozygote (ε4/ε4). The k_*w*_ map from the homozygote (ε4/ε4) displays the lowest k_*w*_ values, which are associated with increased SUVRs of [^11^C]PiB-PET. On the other hand, there were indiscernible differences for QSM among the groups. BBB, blood–brain barrier; PiB, Pittsburgh compound B; QSM, quantitative susceptibility mapping; SUVR, standard uptake value ratio (adapted with permission from Uchida et al., 2022b).

Brain iron accumulation has been proposed as one of the pathomechanisms in neurodegenerative diseases, including Parkinson’s disease (Langkammer et al., 2016; Acosta-Cabronero et al., 2017; Uchida et al., 2019, 2020b), amyotrophic lateral sclerosis (Kwan et al., 2012; Acosta-Cabronero et al., 2018a), Huntington’s disease (Domínguez et al., 2016; van Bergen et al., 2016a), and AD (Acosta-Cabronero et al., 2013; Ayton et al., 2017; Kim et al., 2017; Tiepolt et al., 2018; Gong et al., 2019; Cogswell et al., 2021). QSM can be used to detect abnormal iron deposits in specific affected regions of neurodegenerative diseases, such as in the nigrostriatal system for Parkinson’s disease, the motor cortex for amyotrophic lateral sclerosis, the basal ganglia for Huntington’s disease, and limbic system for AD. Although abnormally high levels of iron are thought to induce free radicals resulting in neuronal loss and clinical symptoms, whether iron deposition is a cause or a result of neurodegeneration remains elusive. The former is supported by clinicoradiological studies revealing iron leakage owing to blood–brain barrier disruption in small vessel diseases (Mikati et al., 2014; Tariq et al., 2018; Uchida et al., 2020a) and subtle blood–brain barrier dysfunction in early stages of Alzheimer’s continuum with the *ε4* allele of *APOE* gene (Figure 1; Yamanaka et al., 2019).

## Relationship between quantitative susceptibility mapping and Alzheimer’s disease pathology

### Altered iron metabolism in Alzheimer’s disease pathogenesis

Altered iron metabolism has been hypothesized to be associated with the pathogenesis of AD (Ayton et al., 2015). Histochemical and histopathological studies have shown evidence of altered iron metabolism and accumulation in AD brain tissues, with iron colocalizing with Aβ aggregates as senile plaques and intracellular hyperphosphorylated tau aggregates as neurofibrillary tangles (Aillaud and Funke, 2022). QSM has been used to study the relationships between cerebral iron load and established biomarkers for AD (Acosta-Cabronero et al., 2013; Ayton et al., 2017; Kim et al., 2017; Tiepolt et al., 2018; Gong et al., 2019; Cogswell et al., 2021). Overall, these findings suggest that magnetic susceptibility in deep gray matter may be a biomarker for AD pathogenesis. Meanwhile, the sensitivity of QSM for the cerebral cortices is insufficient for reliable detection. This is partly due to superficially eroded masking applied and noise levels, such as adjacent to vessels or edges of the brain mask. An advanced multi-scale approach to QSM can improve the ability to detect susceptibility values in the cerebral cortices (Acosta-Cabronero et al., 2018b).

### Association of quantitative susceptibility mapping with Aβ pathology

Senile plaques, which are pathological aggregates of extracellular Aβ proteins, contain iron (Lovell et al., 1998). In an amyloid mouse model of AD, magnetic susceptibility increased over time relative to controls in a longitudinal study, which used a linear mixed effects modeling analysis that incorporated estimates from multiple brain regions (Klohs et al., 2013). Notably, Aβ itself has slightly diamagnetic susceptibility in a phantom experiment (−0.024 to −0.019 ppm) (Gong et al., 2019). Paramagnetic source of β-amyloid plaques *in vivo* is largely attributed to focal iron deposition (Jack et al., 2004). Accordingly, QSM, which is sensitive to the concentration of iron in brain tissues, may play a key role in tracking the progressive pathology of AD and provide a means to measure the efficacy of iron chelation therapy (Crapper McLachlan et al., 1991; Dixon et al., 2012; Liu J. L. et al., 2018; Cummings et al., 2019).

### Association of quantitative susceptibility mapping with tau pathology

Neurofibrillary tangles, which are pathological insoluble aggregates of hyperphosphorylated tau proteins, also contain iron (Good et al., 1992). Susceptibility values of tau protein are diamagnetic as well as Aβ and variable due to echo time (−0.071 to −0.037 ppm) (Gong et al., 2019). In animal models of tau pathology, reactive microglia and astrocytes have been reported to induce neuroinflammation and iron accumulation (Yoshiyama et al., 2007; Maphis et al., 2015). Therefore, QSM may be a sensitive *in vivo* biomarker for these pathological traits. In an analogous model of tau pathology, semi-automatic segmentation of QSM was employed to calculate magnetic susceptibility in gray matter and white matter regions, and it might be useful for detecting early tau pathological changes (O’Callaghan et al., 2017). These QSM protocols could be incorporated into clinical protocols for human AD and other tauopathies that are currently ongoing.

### Association of quantitative susceptibility mapping with neurodegeneration

Based on the ATN system (Jack et al., 2018), biomarkers of neurodegeneration (labeled “N”) include structural MRI, positron emission tomography (PET) with 2-deoxy-2-[fluorine-18]fluoro-D-glucose (^18^F-FDG-PET), and cerebrospinal fluid total tau proteins. In terms of associations between QSM and structural MRI, voxel-based QSM analyses revealed increased susceptibilities of the hippocampus in patients with AD compared to age-matched cognitively normal controls (Acosta-Cabronero et al., 2013; Kim et al., 2017; Kan et al., 2020), whereas voxel-based morphometry revealed atrophic changes of the hippocampus (Matsuda, 2016; Kan et al., 2020). Additionally, a longitudinal study of cognitively normal adults showed that accumulation of iron in the putamen could predict its shrinkage (Daugherty and Raz, 2016). Although less investigated for associations between QSM and the other biomarkers of neurodegeneration, a combined ^18^F-FDG-PET and QSM study in different AD cohorts revealed glucose hypometabolism and brain iron accumulation in the hippocampus, temporal, and parietal lobes (Rao et al., 2022).

### Association of quantitative susceptibility mapping with cognitive decline

Approximately 10–40% of cognitively normal older individuals have evidence of cerebral Aβ deposition (Jansen et al., 2015), which suggests that Aβ alone may not be sufficient for the development of AD symptoms. Histopathological studies have proposed that Aβ and iron colocalize and act synergistically to affect downstream AD pathogenesis (Smith et al., 1997; Gong et al., 2019). Biochemically, Aβ and tau proteins bind ferric iron and reduce it to its redox-active form, ferrous iron, which reacts with hydrogen peroxide to generate reactive oxygen species that lead to ferroptosis pathway (Sayre et al., 2000; Everett et al., 2014; Conrad et al., 2016). Furthermore, a number of clinicoradiological studies emphasize cerebral iron accumulation combined with Aβ and tau proteins to accelerate cognitive decline (van Bergen et al., 2016b; Ayton et al., 2017; Kim et al., 2017; Tiepolt et al., 2018). However, recent whole-brain analyses of QSM with amyloid and tau PET have revealed contradictory evidence, with each pathologic substrate arising independently and in spatially different areas (Cogswell et al., 2021). In voxel-based QSM and amyloid PET analyses, there were clusters in which iron levels were negatively correlated with Aβ deposits, some of which were associated with global cognition (Chen et al., 2020). Further investigations regarding the interactions among iron, Aβ and tau proteins, and cognitive dysfunction are warranted, along with longitudinal studies to determine whether QSM can predict cognitive decline in patients with early stage AD.

### Association of quantitative susceptibility mapping with white matter alteration

Normal white matter regions have negative magnetic susceptibilities due to the presence of myelin, with reference to the cerebrospinal fluid in the ventricle (Wisnieff et al., 2015). Alterations in the magnetic susceptibility of white matter lesions depend on various pathophysiological conditions, including demyelination, ischemia, and expansion of the perivascular space (Sjöbeck et al., 2005). Magnetic susceptibility measurements in white matter using QSM have been shown to be more specifically related to myelin concentration than diffusion tensor imaging (Argyridis et al., 2014). A voxel-based QSM comparison of the whole brain between patients with AD and age-matched cognitively normal controls revealed increased magnetic susceptibilities of the medial temporal lobes in the gray matter and the genu, body, and splenium of the corpus callosum in the white matter (Kan et al., 2020).

## Role of quantitative susceptibility mapping as biomarker for Alzheimer’s disease

### Alternative biomarker for positron emission tomography remains controversial

Several clinicoradiological studies have investigated the relationship between iron and Aβ deposition as detected using QSM and amyloid PET (van Bergen et al., 2016a,^2018^; Ayton et al., 2017; Tiepolt et al., 2018). However, these associations remain controversial, with one study showing no significant association in the cortices (Cogswell et al., 2021) and one that showed a positive or negative correlation that depended on the anatomical brain regions (Chen et al., 2020). In individuals with evidence of cerebral Aβ deposition, higher baseline hippocampal iron levels predict an accelerated longitudinal decline in episodic memory, executive dysfunction, and attention (Ayton et al., 2017).

Compared with amyloid PET, the association between QSM and tau PET has been less investigated. Although some studies have found positive correlations between magnetic susceptibility and tau PET standardized uptake value ratios in the basal ganglia and cortices (Choi et al., 2018; Spotorno et al., 2020; Cogswell et al., 2021), these associations were partly caused by off-target binding of tau PET ligands. Postmortem studies using multiple tau tracers have shown that off-target tau binding is secondary to monoamine oxidase and iron deposition in the presence of inflammation (Harada et al., 2018; Lemoine et al., 2018; Baker et al., 2019).

The extent of elevated magnetic susceptibility in QSM and standardized uptake value ratios in amyloid and tau PET do not overlap, which may imply that more complicated factors contribute to these signal changes. When the anterior hippocampus was segmented into seven layers using high-resolution *ex vivo* MRI, the molecular changes in Aβ and tau protein aggregations had specific effects on the magnetic susceptibilities of AD brain tissues (Zhao et al., 2021). However, layer-specific PET analysis is impractical due to its low resolution.

### Expectations

Numerous concomitant disease processes, including altered iron metabolism, contribute to AD pathogenesis. Proteins such as Aβ and tau that are associated with AD pathology are involved in molecular crosstalk with iron homeostatic proteins (Reed et al., 2009). Furthermore, lipid peroxidation and oxidative stress, hallmark features of ferroptosis, are considered an early event in AD pathogenesis (Praticò and Sung, 2004). From the viewpoint of these pathomechanisms related to perturbations in iron homeostasis, iron itself should be included as pathological biomarker for AD (Masaldan et al., 2019), in addition to the proposed ATN classification system (Jack et al., 2018). Taking account of its presence prior to Aβ and tau aggregates, the possibility of iron chelation therapy is implicated (Crapper McLachlan et al., 1991; Smith et al., 1997; Dixon et al., 2012; Guo C. et al., 2013). With current imaging techniques allowing for *in vivo* quantification of brain iron, Aβ, tau, and neurodegeneration, the efficacy of the disease modifying therapy on these AD pathologies could be more specifically monitored (Borlongan, 2012). An overview of QSM study design and main findings for AD continuum subjects are summarized in Table 2 (Acosta-Cabronero et al., 2013; Hwang et al., 2016; Moon et al., 2016; van Bergen et al., 2016b,^2018^; Ayton et al., 2017; Kim et al., 2017; Meineke et al., 2018; Tiepolt et al., 2018; Chen et al., 2020; Kagerer et al., 2020; Kan et al., 2020; Tuzzi et al., 2020; Cogswell et al., 2021; Ravanfar et al., 2021; Uchida et al., 2022b).

**TABLE 2:** Overview of QSM studies for AD continuum subjects.

Study	Modality	Sample size	Mean age (y) ± SD	Disease severity	Regions of interest	Regions of reference	Associations with established AD biomarkers and cognition
Acosta-Cabronero et al. (2013)	MPRAGE, QSM	AD: 8, HC: 11	AD: 72 ± 6, HC: 70 ± 5	MMSE: AD: 22 ± 4	AMY, CN, GP, HP, PUT, TH, whole brain	Posterior ventricular region	No associations of QSM with HP atrophy
van Bergen et al. (2016b)	Amyloid PET, fMRI, QSM	MCI: 15, HC: 22	MCI: 75.27 ± 7.63, HC: 71.91 ± 5.25	MMSE: MCI: 28.61 ± 1.65	AMY, CN, EC, GP, HP, NAc, neocortices, PUT, TH	Frontal central CSF	Positive associations of QSM with Aβ deposition in medial prefrontal cortex in MCI group
Moon et al. (2016)	QSM	AD: 27, HC: 18	AD: 78.63 ± 8.11, HC: 46.89 ± 14.69	MMSE: AD: 14.70 ± 5.81	CN, GP, PUL, PUT	NA	No associations of QSM with age and severity of cognitive deficits
Hwang et al. (2016)	QSM	AD: 18, MCI: 18, HC: 18	AD: 69. 9 ± 9.81, MCI: 66.9 ± 5.51, HC: 65.2 ± 6.41	MMSE: AD: 17.56 ± 3.5, MCI: 27.61 ± 2.17	HP, PUT, whole brain	Posterior ventricular region	Increased QSM values of whole white matter in AD subjects
Ayton et al. (2017)	Amyloid PET, QSM	AD:19, MCI: 17, HC: 64	Aβ+: 76.4 ± 1.0, Aβ-: 74.0 ± 0.9	NA	Cingulate, CN, HP, neocortices	Middle frontal white matter region	Colocalization of QSM with Aβ deposition in frontal, temporal, and occipital lobes in MCI group, inverse associations of QSM with cognition in Aβ+ subjects
Kim et al. (2017)	MPRAGE, QSM	AD: 19, MCI: 19, HC: 19	AD: 69.79 ± 10.27, MCI: 65.95 ± 6.75, HC: 65.37 ± 6.29	MMSE: AD: 17.37 ± 3.42, MCI: 27.63 ± 2.11	AMY, GP, HP, neocortices, PC, PUL, PUT, TH,	Posterior ventricular region	Increased QSM values of neocortices in AD subjects
Tiepolt et al. (2018)	Amyloid PET, QSM	AD: 10, HC: 10	AD: 74.1, HC: 67.1	MMSE: AD: 23.6 ± 7.3	GP, neocortices, PUT	CSF	No associations of QSM with amyloid-PET
van Bergen et al. (2018)	Amyloid PET, QSM	Elderly: 116	74.81 ± 7.52	MMSE: Elderly: 28.99 ± 1.10	Whole brain	Deep frontal white matter	Positive associations of QSM with amyloid-PET in CN, GP, PUT, and neocortices
Meineke et al. (2018)	QSM	AD: 6, MCI: 8, HC: 10	AD: 58 ± 6, MCI: 63 ± 6, HC: 59 ± 7	MMSE: AD: 19.2 ± 3.2, MCI: 25.6 ± 2.1	CN, GP, HP, PUT, TH	Corpus callosum	Increased QSM values of CN and PUT in AD subjects
Chen et al. (2020)	Amyloid PET, QSM	Elderly: 150 (PET: 97)	Elderly: 69 ± 8 (PET: 71 ± 6)	GCS: Elderly: 0.31 6 ± 0.57 (PET: 0.33 ± 0.54)	AMY, Cingulate, CN, EC, GP, HP, neocortices, PUT	CSF	Inverse associations of QSM with cognition independent of amyloid-PET in HP
Kan et al. (2020)	MP-QSM	AD: 38, HC: 19	AD: 80 ± 6, HC: 71 ± 5	NA	Whole brain	CSF	Increased QSM values of AMY, CN, and HP in AD subjects
Kagerer et al. (2020)	Amyloid-PET, BOLD, QSM	APOE4+: 18, AOE4–: 51	APOE4+: 66.28 ± 5.29 APOE4–: 66.04 ± 7.87	MMSE: APOE4+: 29.12 ± 1.58, APOE4−: 29.4 ± 0.89	DMN	Deep frontal white matter	Positive associations of QSM with DMN activity that in APOE4+ subjects
Tuzzi et al. (2020)	QSM	AD: 2, HC: 2	NA	NA	Frontal cortex	Whole brain	Increased QSM values of frontal cortex in AD subjects
Cogswell et al. (2021)	Amyloid-PET, MPRAGE, Tau-PET, QSM	MCI: 56, AD: 69, HC: 296	AD: 68 (61–77), MCI: 77 (72–86), HC: 69 (59–76)	STMS: AD: 22 (18–28), MCI: 32 (29–33), HC: 37 (36–38)	Cingulate, CN, GP, neocortices, PUT, RN, SN, STN, TH	Frontal white matter	Positive associations of QSM with amyloid PET in pallidum and putamen, tau PET in pallidum, and lower cortical gray matter volume in medial temporal lobe
Uchida et al. (2022b)	Ingenia, Philips	APOE4/4: 20, APOE3/4: 22, APOE3/3: 24	APOE4/4: 27.6 ± 2.7, APOE3/4: 27.6 ± 2.5, APOE3/3: 28.0 ± 2.1	MMSE: APOE4/4: 72.8 ± 5.6, APOE3/4: 72.1 ± 6.1, APOE3/3:71.7 ± 6.2	Cingulate, neocortices, PC	CSF	Positive associations of QSM with amyloid PET in frontal lobe independent of APOE4 dose, inverse associations of QSM with executive function independent of APOE4 dose

AD, Alzheimer’s disease; AMY, amygdala; APOE, apolipoprotein E; BOLD, blood oxygen level dependent; CSF, cerebrospinal fluid; CN, caudate nucleus; DMN, default mode network; EC, entorhinal cortex; GCS, global cognitive composite score; GP, globus pallidus; HC, healthy control; HP, hippocampus; MCI, mild cognitive impairment; MMSE, mini-mental state examination; MP-QSM, magnetization-prepared spoiled turbo multiple gradient echo sequence with inversion pulse for QSM; MPRAGE, magnetization-prepared rapid gradient-echo; NA, not applicable; NAc, nucleus accumbens; PC, precuneus; PET, positron emission tomography; QSM, quantitative susceptibility mapping; RN, red nucleus; SD, standard deviation; SN, substantia nigra; STMS, short test of mental status; STN, subthalamic nucleus; TH, thalamus.

**FIGURE 2:**
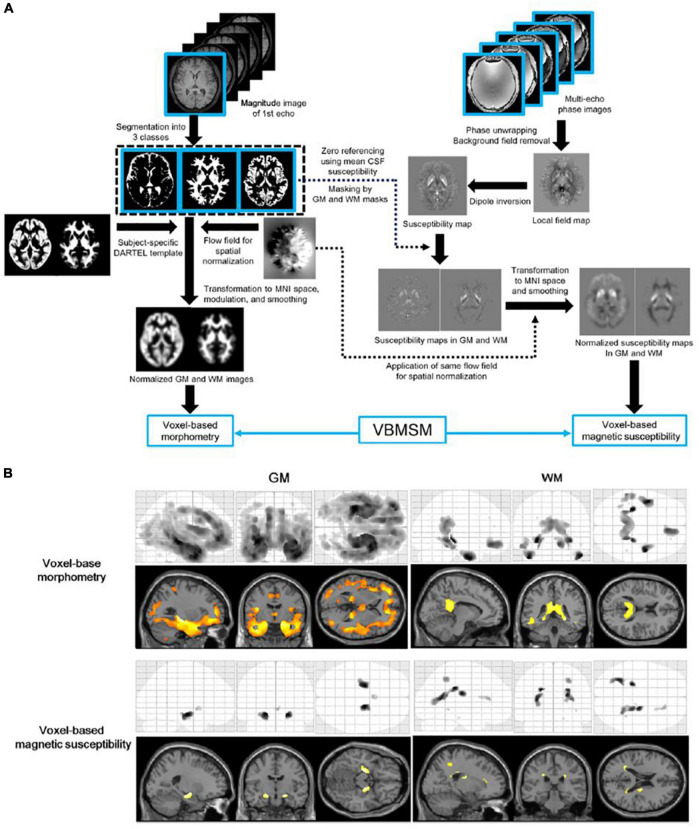
Diagram of voxel-based morphometry and magnetic susceptibility analyses **(A)** and results of the voxel-based analyses **(B)**. The top of the left-hand panel shows the procedure of the voxel-based morphometry analysis. The top of the right-hand panel shows the procedures of the susceptibility estimation and spatial normalization of the map for the voxel-based magnetic susceptibility analysis. The bottom panel shows the results of voxel-based morphometry and magnetic susceptibility comparisons between elderly volunteers and patients with Alzheimer’s disease. A corrected *P*-value of < 0.05 with the family-wise error correction was applied as the threshold to detect regional volume decreases and susceptibility increases in the Alzheimer’s disease group. GM, gray matter; VBMSM, voxel-based magnetic susceptibility and morphometry; WM, white matter (adapted with permission from Kan et al., 2020).

Voxel-based morphometry and QSM analyses are useful for mapping the landscape of whole-brain volume and magnetic susceptibility changes in patients with AD (Ashburner and Friston, 2000; Acosta-Cabronero et al., 2013; Kim et al., 2017). A magnetization-prepared spoiled turbo multiple gradient echo sequence has been developed to simultaneously acquire 3D T1-weighted structural and multi-echo phase images for voxel-based morphometry and QSM analyses (Kan et al., 2020). The key advantage of this technique is that any image registration between these images prior to spatial normalization is unnecessary, as these datasets have exactly the same geometry (Figure 2).

**FIGURE 3:**
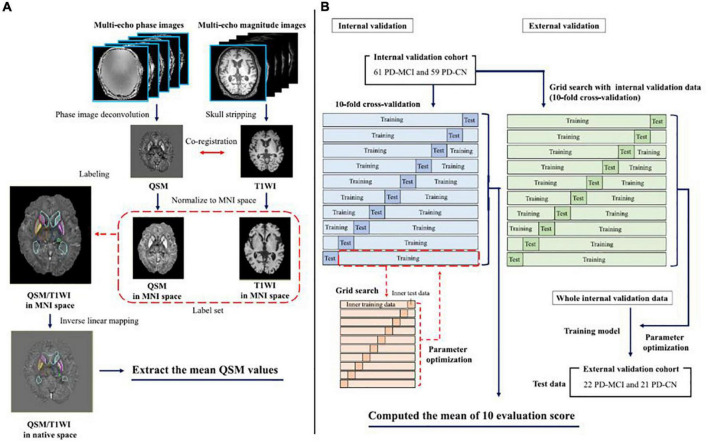
The left-hand panel shows the pipeline for the multi-atlas approaches for each individual QSM/T1WI image through the MRICloud platform (https://mricloud.org/) **(A)**. The right-hand panel shows the pipeline for developing the machine learning-based models **(B)**. MNI, Montreal Neurologic Institute; PD-MCI, Parkinson’s disease with mild cognitive impairment; PD-CN, Parkinson’s disease with normal cognition; QSM, quantitative susceptibility mapping; T1WI, T1-weighted image (adapted with permission from Shibata et al., 2022).

Atlas-based analysis, which can help generate universal and sharable susceptibility measures in a biologically meaningful set of anatomical structures, is also useful (Lim et al., 2013). Moreover, the multi-atlas label-fusion method for automated segmentation of QSM images has been developed as a more accurate quantification tool for determining the magnetic susceptibilities of individuals (Li et al., 2019). Figure 3 shows a machine learning model trained with the extracted magnetic susceptibilities using the multi-atlas label-fusion method to detect early cognitive impairments (Shibata et al., 2022).

More advanced QSM techniques should be highlighted: R2* relaxometry analysis combined with QSM can distinguish microstructural changes of white matter demyelination from iron deposition, thereby providing a sensitive and biologically specific measure for white matter lesions (Kan et al., 2022). Recent breakthroughs in small vessel imaging within the central nervous system, such as venous oxygen saturation and blood–brain barrier function using QSM techniques, are promising biomarkers in research and clinical settings for AD (Uchida et al., 2020a,b).

### Limitations

One of the major limitations of the magnetic susceptibility measured by QSM is its non-specific nature. In AD brain research, the contrast to the surrounding brain tissues is considered to be caused mainly by iron deposition; however, it can be caused by other substances, such as calcium, lipids, and myelin (Li et al., 2011; Deistung et al., 2013). Current QSM approaches are unable to identify the chemical configurations underlying abnormal magnetostatic behaviors. Another is that multiple iron containing species may interact differently with Aβ and tau proteins (Sayre et al., 2000; Everett et al., 2014). It remains unclear whether QSM is equally sensitive to iron in different states, as each species of iron may have a different intrinsic magnetic susceptibility. These complexities of the QSM technique could result in experimental variability in the associations of magnetic susceptibilities with PET signals and explain some of the seemingly contradictory findings in different populations. Precise relationships between QSM and established AD biomarkers should be elucidated in the near future by applying ultra-high field acquisition protocols (Alkemade et al., 2020; Tuzzi et al., 2020) and machine learning algorithms (Kim et al., 2020).

## Conclusion

The QSM technique provides a sensitive and biologically specific contrast of magnetic susceptibilities. Hence, it can be used for *in vivo* characterization in accordance with tissue magnetic susceptibilities, ranging from common applications, such as cerebral iron deposition, to more recent applications, such as assessment of impaired myelination, quantification of venous oxygen saturation, and measurement of blood–brain barrier function. Therefore, the acquisition sequence for post-processing susceptibility maps should be included in routine applications due to its high-throughput computing nature with important implications. We conclude that QSM has the ability to provide pathophysiological information on brain tissue properties and the potential to measure the efficacy of novel therapeutics in clinical settings for AD.

## Author contributions

YU: conceptualization, investigation, data curation, writing – original draft, and funding acquisition. HK: conceptualization, data curation, and writing – review and editing. KS: data curation and writing – review and editing. KO: supervision and writing – review and editing. NM: conceptualization, supervision, and writing – review and editing. All authors contributed to the article and approved the submitted version.
